# Iodine stimulates estrogen receptor singling and its systemic level is increased in surgical patients due to topical absorption

**DOI:** 10.18632/oncotarget.20633

**Published:** 2017-09-04

**Authors:** Shaohua He, Bingchan Wang, Xiyi Lu, Suyu Miao, Fengming Yang, Theodore Zava, Qiang Ding, Shijiang Zhang, Jiayin Liu, David Zava, Yuenian Eric Shi

**Affiliations:** ^1^ Departments of Oncology, The First Affiliated Hospital of Nanjing Medical University, Nanjing, China; ^2^ ZRT Laboratory, Beaverton, Oregon, USA; ^3^ Departments of Surgery, The First Affiliated Hospital of Nanjing Medical University, Nanjing, China; ^4^ Departments of Cardiology, The First Affiliated Hospital of Nanjing Medical University, Nanjing, China; ^5^ Departments of Obstetrics and Gynecology, The First Affiliated Hospital of Nanjing Medical University, Nanjing, China; ^6^ State Key Laboratory of Reproductive medicine, Nanjing Medical University, Nanjing, China

**Keywords:** iodine, ER-α, breast cancer

## Abstract

Iodine is crucial for thyroid hormone production. However, recent epidemiologic studies have shown that breast cancer patients have an elevated risk of developing thyroid cancer and vice versa. A notable finding in this study is that iodine stimulated the transcriptional activity of estrogen receptor-α (ER-α) in breast cancer cells. Iodine stimulated expression of several ER-α regulated gene including *PS2*, *Cathepsin D*, *CyclinD1*, and *PR* both *in vitro* and in nude mice, which is consistent with its stimulation of both anchorage-dependent and -independent growth of ER-α positive breast cancer cells and the effect to dampen tumor shrinkage of MCF-7 xenograft in ovariectomized nude mice. Analyses of clinical urine samples from breast cancer patients undergoing surgery demonstrated that urinary iodine levels were significantly higher than that in controls; and this increased level is due to the antiseptic use of iodine during breast surgery. The present study indicates that excess iodine intake may be an unfavorable factor in breast cancer by stimulation of ER-α transcriptional activity.

## INTRODUCTION

It is well-known that the iodine plays a key role in thyroid hormone production and the maintenance of thyroid gland epithelial integrity [1]. The thyroid gland accumulates iodine by a active transport mechanism mediated by sodium iodide symporter (NIS) [2], a glycoprotein expressed at the basolateral surface of the follicular cells [3]. NIS is a Na+/K+ ATPase-dependent active iodide (I^−^) transport [3–5]. In addition to thyroid, NIS also mediates iodide absorption by the stomach, salivary gland, and lactating mammary gland [2, 6].

Incidences of thyroid related diseases including hyperthyroidism, autoimmune thyroiditis, and thyroid cancer have been elevated since iodized salt became mandated in 1997 [7, 8]. Clinical studies indicate that thyroid cancer patients undergoing ^131^I treatment have an increased incidence of developing secondary malignancies of breast and stomach, which are capable of iodine uptake by expression of NIS [6, 9, 10]. It has been also reported that the serum and urinary iodine levels are significantly higher among patients with gastric cancer, particularly in the late stage of gastric cancer [11].

For several decades, research suggests that some breast diseases may be considered iodine deficiency diseases. Evidence supporting this hypothesis include: 1) iodine-rich seaweed exhibits an anti-cancer effect on breast cancer cells and on animal model [12]; 2) adding seaweed to rats’ food delays the onset and number of rat mammary tumors [13, 14]; 3) for women experiencing painful breasts with fibrocystic disease, iodine improved symptoms [15]; and 4) iodine consumption by Americans has dropped 50% since the 1970s as breast cancer rates have risen [16]. By contrast, Japanese women consume 25 times more dietary iodine than North American women and have lower breast cancer rates [17, 18]. However, the increased incidences of both breast cancer and thyroid cancer since the use of iodized salt became mandated have raised some concerns challenging this consensus. Several recent epidemiologic studies have shown that breast cancer patients have an elevated risk of developing thyroid cancer and vice versa [19, 20]. The risk of thyroid cancer following breast cancer was increased by 31%–73% and that of breast cancer following thyroid cancer by 21%–89% [20]. Furthermore, the presence of breast cancer in thyroid cancer patients aged 40–50 years was found to be three times more likely than in age-matched controls [21]. Furthermore, studies indicate that the incidence of cancer, especially cancer of the breast and stomach, is higher in patients treated with first-line therapy radioactive iodine (RAI) for hyperthyroidism [22].

Approximately 30% of total iodine in the body is concentrated in the thyroid gland; the rest of the iodine is present in other tissues [18]. Iodine has been shown to be highly absorbed in mammary glands during pregnancy and lactation [18, 23]. In a pioneering study, Tazebay demonstrated that NIS is expressed in 87% of human invasive breast cancer cases, compared with 23% of surrounding non-cancerous breast tissue [4]. In contrast, NIS was not detected in normal healthy breast. Various groups have reported NIS over-expression in breast cancer samples [4, 24–26]. Interestingly, a recent study demonstrated that the expression of NIS on breast cancer cells is positively associated with ER-α expression [25]. The possible involvement of iodine in hormone-responsive cancers of breast and the co-expression of NIS and ER prompted us to explore the potential role of iodine in cellular response to estrogen. In the present study, we evaluated the biological functions of iodine on stimulation of transcriptional activity of ER-α in human breast cancer cells *in vitro* and *in vivo*. We also analyzed the urinary iodine levels in the breast cancer patients undergoing surgery.

## RESULTS

### Iodine stimulated transcriptional activity of ER-a

**Figure 1:**
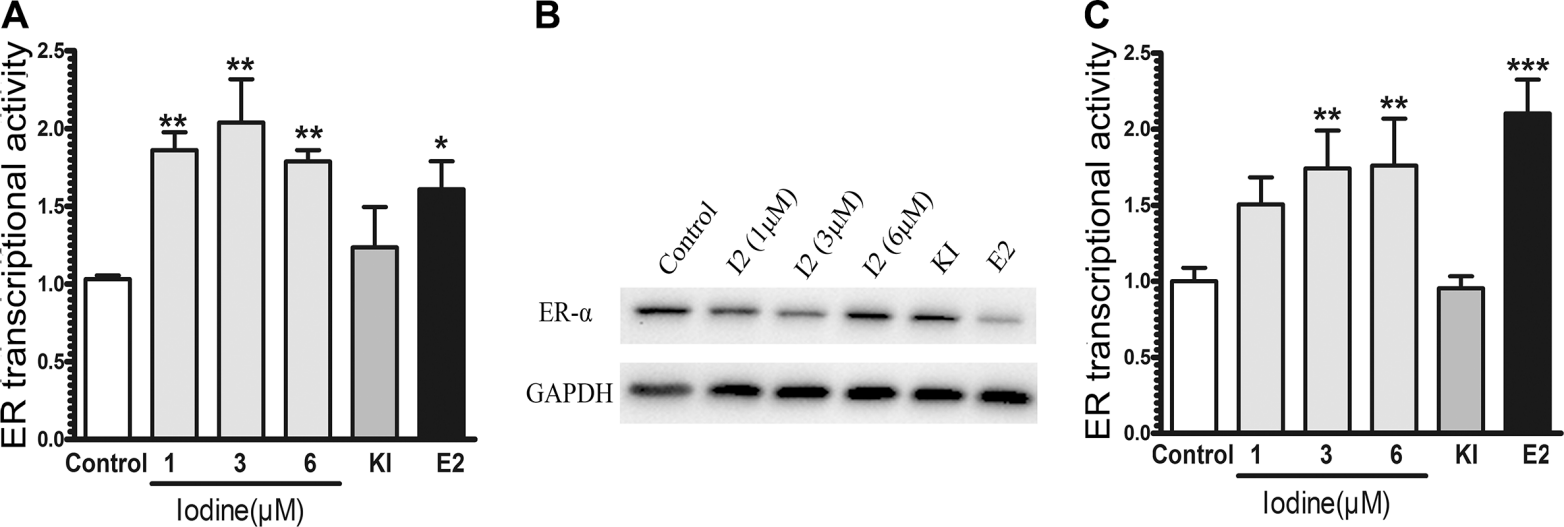
Stimulation of ER signaling MCF-7 (**A**) and ER-α stably transfected MDA-MB-231 (**C**) cells were transiently transfected with pERE4-Luc as well as control reporter pRL-SV40-Luc. After transfection, cells were cultured in the estrogen-free conditioned medium for 3 days as described in Materials and methods, treated with iodine (1, 3, 6 μM), E2 (1 nM), and KI (5 mM) for 24 h before the promoter activities were determined by measuring the dual luciferase activity. The ERE reporter luciferase activity was normalized against the control renilla luciferase activity to correct for transfection efficiency. All values were presented as the fold induction over the control luciferase activity in the non-treated control cells, which was taken as 1. The numbers represent means ± s.d. of three cultures. (**B**) Western analysis of ERα expression. MCF-7 cells were treated with iodine (1, 3, 6 μM), E2 (1 nM), and KI (5 mM) for 48 hours, protein isolated, subjected to Western analysis, and normalized with GAPDH. ^**^ stand for *p* < 0.01;^*^ stand for *p* < 0.05.

Estrogen response is mainly mediated by nuclear receptor family of transcription factors, ER-α and ER-β, and a membrane-bound ER-α36 [27, 28]. Since ER-α is the major estrogen receptor in mammary epithelia, we measured the effect of iodine on modulating the transcriptional activity of ER-α in human breast cancer cells. MCF-7 cells were transiently transected with estrogen-responsive luciferase reporter plasmid. Iodine significantly stimulated the ER-α activity, with a 2.1-fold increase in ER-α transcriptional activity (Figure 1A). The effect of potassium iodide (5 mM) on ER-α activity did not show significant difference compared to the control group, which is consistent with the function of KI as an agent that maintains sufficient molecular iodine concentration. As a positive control, E2 stimulated ER-α transcriptional activity 1.7-fold. As expected, the treatment of the cells with E2 resulted in a significant decrease in ER-α level (Figure 1B). Iodine treatments (1 μM, 3 μM, 6 μM ) slightly decreased the expression of ER-α protein with a maximum inhibitory effect occurring at 3 μM (Figure 1B). We also investigated the effect of iodine on the transcriptional activity of ER-α in ER-α negative MDA-MB-231 cells (Figure 1C). A significant stimulation of ER-α signaling by iodine was observed in MDA-MB-231 cells when the cells were cotransfected with ER-α and estrogen-responsive luciferase reporter constructs.

**Figure 2:**
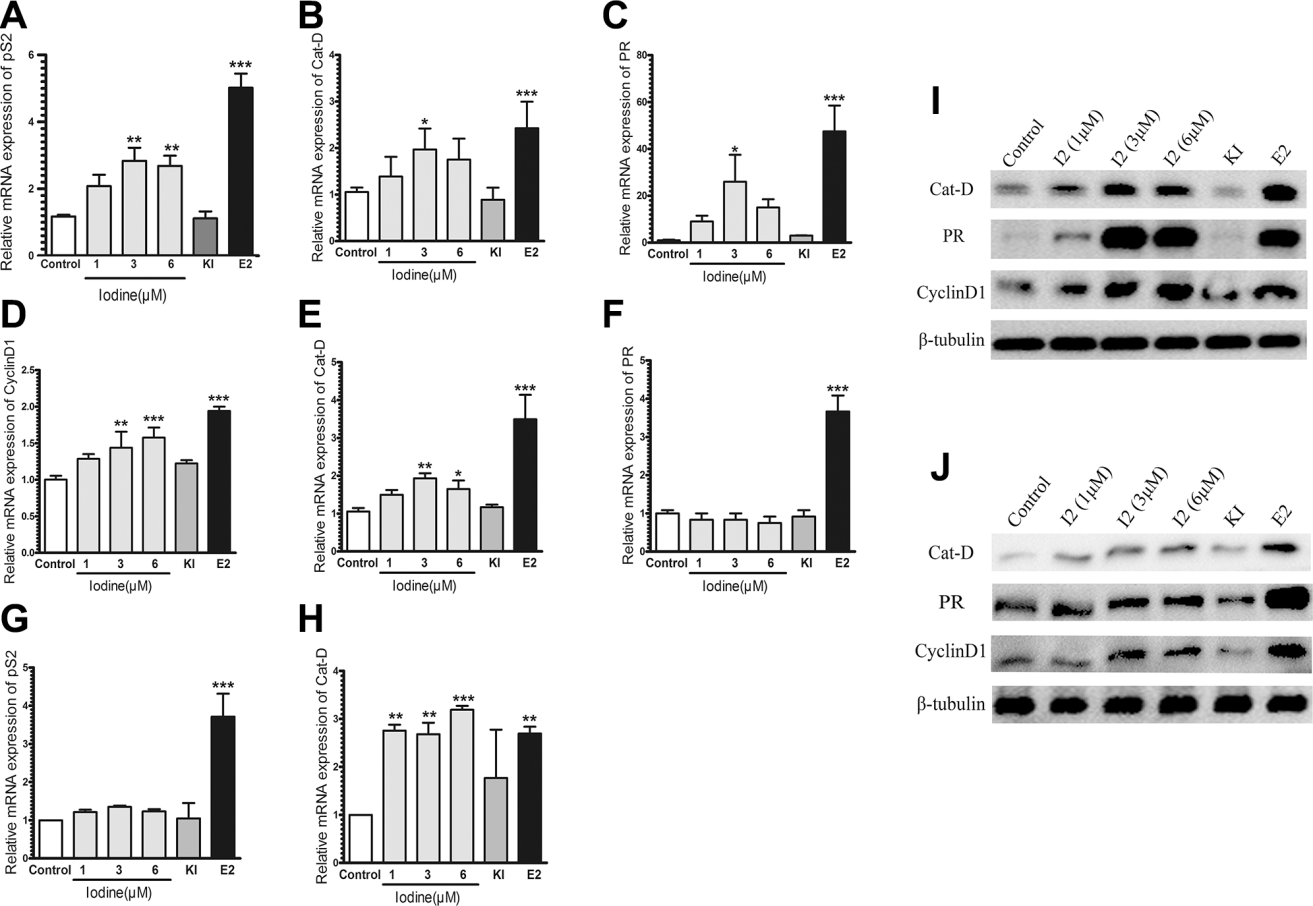
(**A**–**F**) Quantitative RT-PCR analysis of PR, PS2, Cyclin D1, and Cat-D expression. RNAs were isolated MCF-7 (A–C), T47D (D–F), and ER-α transfected MDA-MB-231 (**G**–**H**) cells and subjected to real-time PCR analysis. Relative expressions PS2, PR, Cyclin D1 and Cat-D gene in the cells treated either with E2 (1 nM), Iodine (1–6 μM), and KI (5 mM) were calculated in comparison to that from non-treated cells, which was taken as 1 and regarded as control. All the other values were expressed as a percentage of the control. The human β tubulin gene was used as endogenous control. The numbers represent the means ± s.d. of triplicate RNA samples. ^***^ stand for *p* < 0.001; ^**^ for *p* < 0.01; ^*^ for *p* < 0.05. (**I**–**J**) Western analysis of Cat-D, PR, and Cyclin D1 in MCF-7 (G) and T47D (H) cells. Cells were treated with either Iodine, or E2 (1 nM), or KI (5 mM) for 48 hours, protein isolated, and subjected to Western analysis.

Consistent with the increased transcriptional activity of ER-α in MCF-7 cells, iodine also stimulates E2-regulated genes of *PS2*, *Cathepsin D* (*Cat-D*), and *PR* (Figure 2A–2C). Transcription of *PS2, Cat-D,* and *PR* were increased 2.6-fold, 2-fold, and 17-fold in the iodine (3 uM) treated cells vs. control cells, respectively. At the same conditions, treatment of the cells with E2 (1 nM) resulted in a 4.3-fold, 2.6-fold, and 30-fold increase in expression levels of *PS2, Cat-D,* and *PR*, respectively. We also investigated the effect of iodine on the transcriptional activity of ER-α in T47D (Figure 2D–2F) and ER-α stably transfected MDA-MB-231cells (Figure 2G–2H). As expected, the transcriptional activity of ER-α in T47D cell is much weaker than that in MCF-7 cells. Treatment of T47D cells with E2 stimulated a 2-fold and 3.5-fold increase in *CyclinD1* and *Cat-D*, respectively. Iodine treatment increased the expression levels of CyclinD1 and *Cat-D* a 1.4-fold and 1.9-fold. However, the expression levels of PR were not increased after addition of different concentrations iodine but had 4-fold increase when treated with E2 (Figure 2F). A significant iodine-induced 3-fold increase in Cat-D expression (Figure 2H) and a slight increase in PS2 expression (Figure 2G) was also observed in ER-α transfected MDA-MB-231 cells.

The effect of iodine on protein levels of PR, Cat-D, and CyclinD1 in both of MCF-7 and T-47D cells was investigated. Treatment of MCF-7 cells with iodine significantly up-regulated protein expression of and PR, Cat-D, and CyclinD1 (Figure 2J). Expression levels of Cat-D and CyclinD1 were also increased in T-47D cells after iodine treatment (Figure 2K), but the extent of up-regulation of PR in iodine treated cells was much weaker than in MCF-7 cells.

### Growth stimulation by iodine

**Figure 3:**
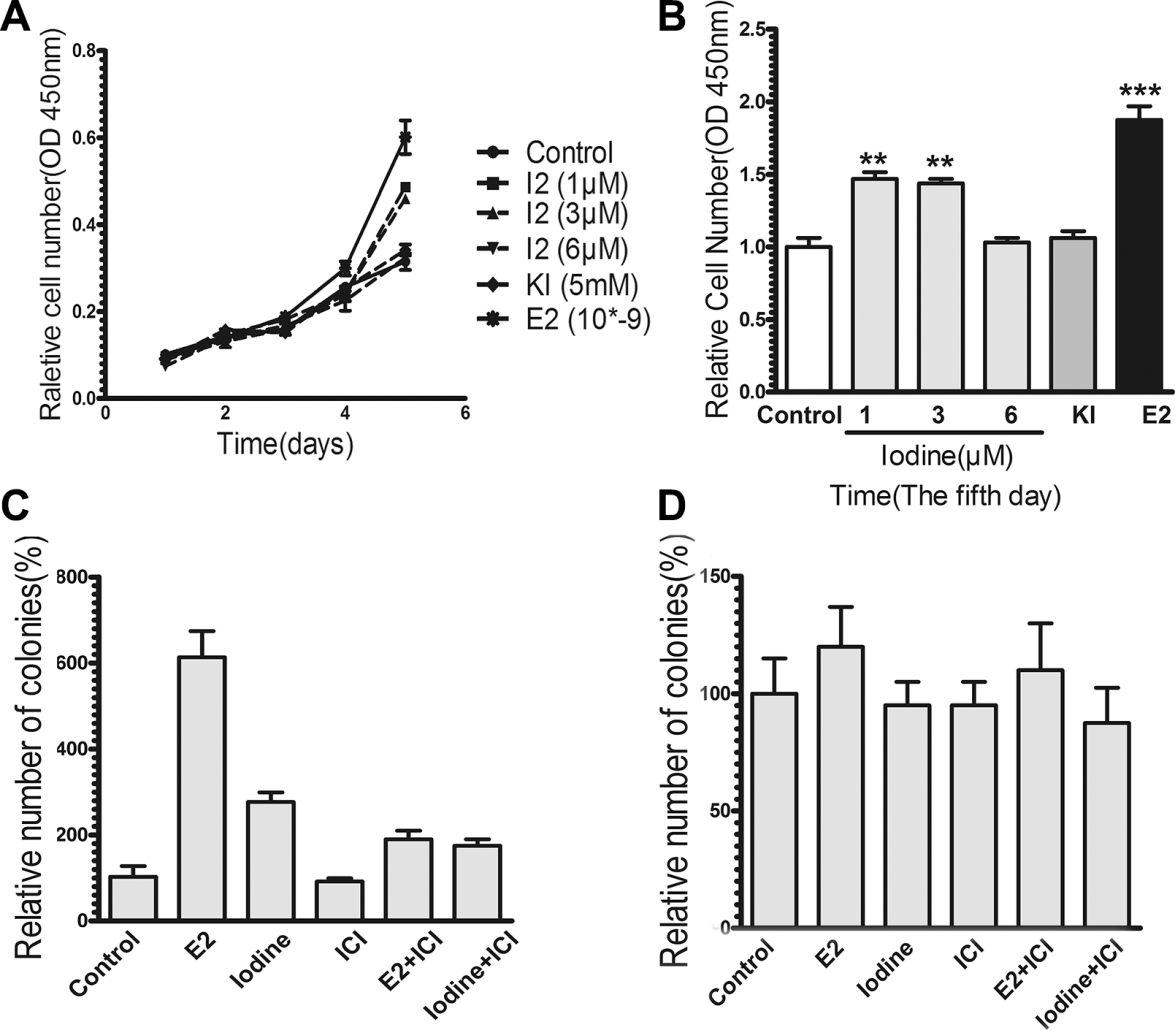
Effects of iodine on cell growth For all experiments, cells were cultured and synchronized in the estrogen-free conditioned medium for 3 days before the treatments. (**A**–**B**) Stimulation of cell proliferation by iodine. Cells were treated with or without iodine (1,3,6 μM) for 5 days. As a positive control, cells were also treated with 1 nM E2. Cell proliferation was measured by Cell Counting Kit-8. Data are means of three cultures. (B) Stimulations of cell growth were calculated in comparison to that from non-treated cells, which was taken as 1 and regarded as control. (**C**–**D**) Effect of iodine on soft agar colonies formation of MCF-7 (C) and MDA-MB-435 (D) cells. Approximate 2000 cells were cultured into the top layer soft agar and treated with or without 3 μM iodine, 1 nM E2, 1 μM ICI, or combination with ICI. The number of colonies was counted after 2 weeks of plating using a Nikon microscope at ×100 amplification. Triplicate wells were assayed for each condition. Statistical comparison of colony formation for E2 or iodine-treated MCF-7 cells relative to control non-treated cells indicates *P* < 0.01.

We analyzed the effect of iodine on the growth of breast cancer cells. As shown in Figure 3A–3B, treatment of MCF-7 cells with iodine stimulated proliferation 1.6-fold and 1.5-fold at the concentrations of 1 uM and 3 uM, respectively. At the higher concentration of 6 uM, iodine has no effect. Potassium iodide (5 mM) has no effect on cell proliferation, which is consistent with previous results. Treatment of MCF-7 cells with E2 stimulated cell proliferation 2-fold over controls.

We studied the effect of iodine on anchorage-independent growth. Soft agar colony assays demonstrated that the anchorage-independent growth of MCF-7 cells was stimulated by both E2 and iodine (Figure 3C). To determine whether the stimulatory effect of iodine on cell growth is mediated by ER-α, we first investigated the effect of the antiestrogen ICI 182,780. ICI 182,780 significantly blocked both E2- and iodine-stimulated colony formations. Secondly, we studied effects of iodine on colony formation of ER-α-negative MDA-MB-435 cells. As shown in Figure 3D, neither E2 nor iodine had an effect on anchorage-independent cell growth. These data indicate that iodine-stimulated cell growth is mediated by ER-α.

### Urine levels of iodine from breast cancer patients, healthy women, and women undergoing surgery for endometriosis

**Figure 4:**
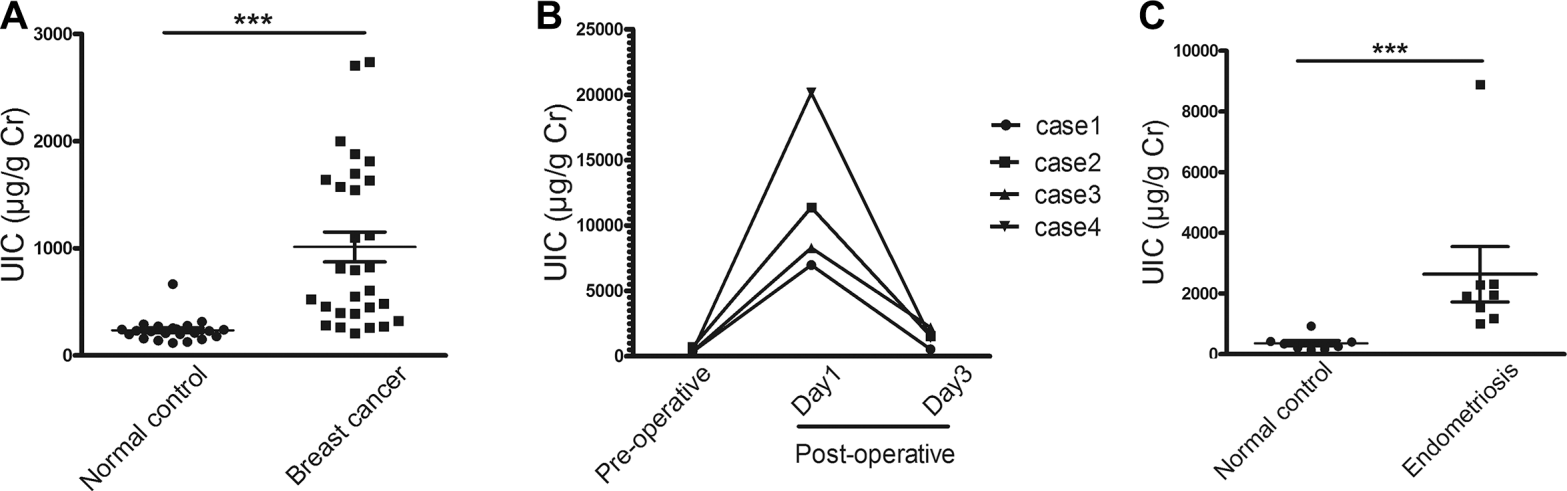
Analysis of urinary iodine concentrations (UIC) of breast cancer patients, healthy women, and the women undergoing surgery for endometriosis (**A**) UIC of patients with histological proved breast cancer and normal healthy controls. Urine samples were collected from 29 breast cancer patients one day following operation (median age 38, range 24 to 46) and 22 normal healthy controls (median age 42, range 32 to 45). All breast cancer patients have topical antiseptic application of Povidone-iodine (PVP-1) before operation. To normalize UIC, iodine levels were expressed as micro gram of iodine per gram of creatinine (^***^, *p* < 0.0001). (**B**) Time-course analysis of UIC in 4 breast cancer patients during operation. Urine samples from preoperative day 1, postoperative day 1, and postoperative day 3 were collected and subjected to analyses. (**C**) UIC of patients with endometriosis and normal healthy controls. Urine samples were collected from 8 patients one day following operation and 8 healthy women. Difference shown as means ± SD, ^***^p < 0.001.

Since iodine stimulates ER-α transcriptional activity, we studied the clinical relevance of iodine on breast cancer patients vs. normal healthy women. As a pilot study, we analyzed urinary iodine levels of 29 patients with histologically proven breast cancer and 22 normal healthy controls. While the mean urinary iodine to creatinine ratio in the control group is 235 ± 110.2 μg/g creatinine, a robust increase in the breast cancer group was observed with a mean ratio of 1012.5 ± 752.2 μg/g creatinine, which is more than four times higher than in healthy controls (*p* < 0.0001) (Figure 4A).

Given the fact that the study population came from the same geographic region and consumed approximately similar amounts of iodine, it was of interest to determine whether the high level of iodine in post-surgical breast cancer patients might play a role in breast cancer incidence or if the elevated iodine is derived from other sources. Because the urine samples from breast cancer patients were collected at day 1-2 days post surgery, we thought that the high level iodine in breast cancer patients might come from topical application of antiseptic Povidone-iodine (PVP-I) during surgery. To determine the possible source of the iodine we tested the urine iodine content in four breast cancer patients and collected urine samples before and at the 1st and 3rd day post-operation. The results clearly demonstrate that the iodine is robustly increased at 1st day and back to approximately original levels at the 3rd day post-operation (Figure 4B). As a control for breast cancer patients, we also compared urine iodine levels from women undergoing laparoscopic surgery for endometriosis and from healthy controls. Similar to what we observed in breast cancer patients, the urine iodine level from endometriosis patient post surgery is significantly increased, reaching a 6.7-fold increase over healthy controls (Figure 4C). Our data indicate that there is a robust increase in systemic urinary iodine level in patients following the topical application of PVP-I during operation

### *In vivo* effects of topical application of iodine on expression of estrogen-regulated genes and tumor growth

**Figure 5:**
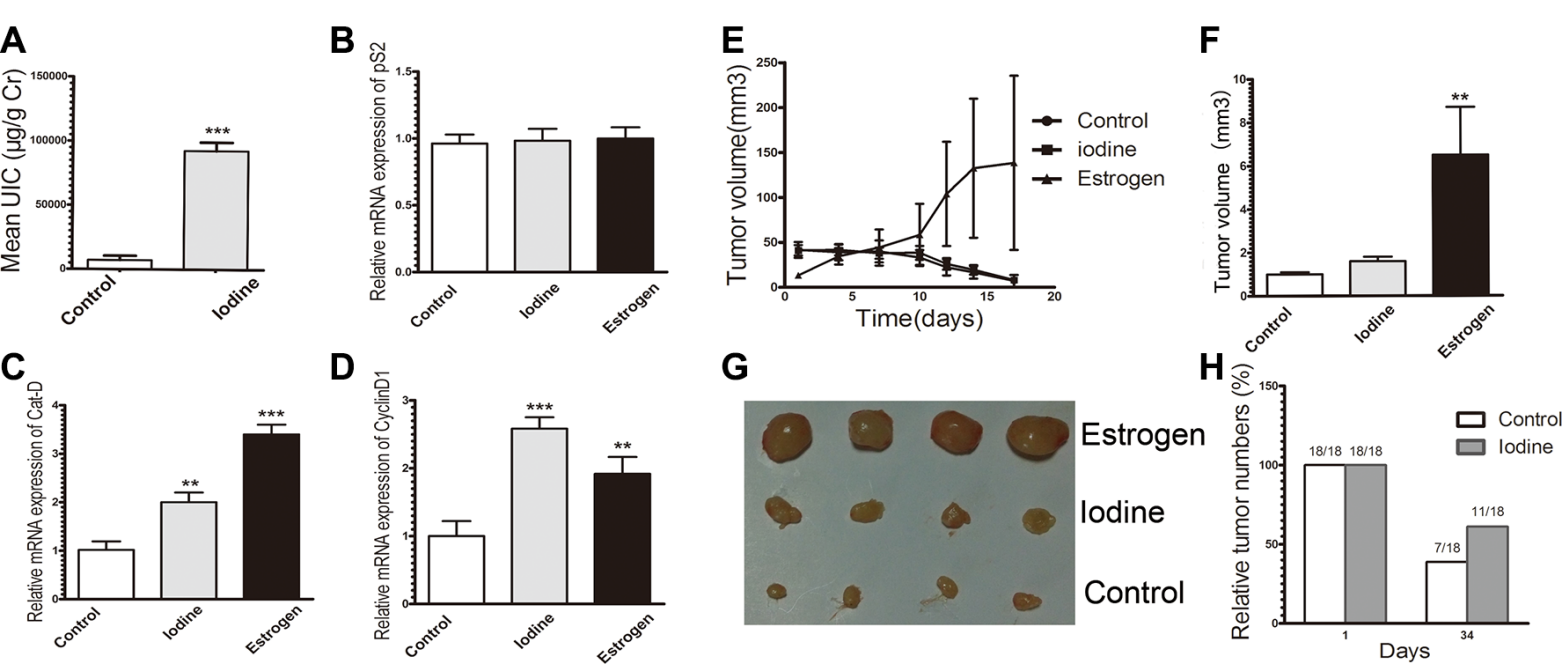
(**A**–**D**) Stimulation of ERα signaling in tumor xenograft. MCF-7 tumor cell injection into ovariectomized athymic mice, estrogen supplement, and topical application of Iodine Tincture were descried in Materials and Methods. There were 9 mice in each group and each mouse received two injections, one on each side. (A) Average urine iodine concentrations of representative 3 control and 3 Iodine Tincture-treated mice. Urine samples were collected at day 15 after tumor cell injection and subjected to iodine analysis. (B–D) Expression of estrogen regulated genes of pS2 (B), Cyclin D1 (C), and Cat-D (D) of tumors. RNA samples were isolated from tumors of 3 non-treated and 3 Iodine Tincture treated mice, and subjected to quantitative RT-PCR analysis (**E**–**H**) Effects of iodine on MCF-7 tumor regression. Each point represents the mean of tumors ± SE. (E) Growth of MCF-7 tumors in control, iodine-treated, and E2 supplement mice. All mice were sacrificed at day 34 after tumor cell inoculation. (F) Relative MCF-7 tumor sizes. Tumor sizes were measured at 34-days following tumor cell inoculation. Stimulations of tumor growth were calculated in comparison to that from control non-treated mice, which was taken as 1 and regarded as control. Statistical comparison for tumor size in E2 treated mice relative to control mice indicates ^*^P < 0.001. (G) Representative MCF-7 tumor xenograft sizes at 34-days when tumor-bearing mice sacrificed. (H) Tumor incidence MCF-7 xenograft in control and iodine treated mice at day 1 and day 34.

To investigate the *in vivo* effect and to imitate the clinical observation, we determined the effect of topical application of iodine on the established MCF-7 tumor in ovariectomized athymic nude mice. We first analyzed the effect of topical application of Iodine Tincture on iodine absorption and on estrogen-regulated genes in MCF-7 tumor. Topical application of Iodine Tincture increased urine levels of iodine, suggesting an efficient skin absorption in nude mice (Figure 5A). Although *pS2* expression on both iodine and estrogen treated groups show no significant difference compared to control levels (Figure 5B), expression levels of *CyclinD1* and *Cat-D* was significantly higher in the iodine or estrogen treated nude mouse (*P* < 0.05) when compared with the average expression levels of control group (Figure 5C–5D). These data suggest that skin-absorbed iodine has an estrogenic effects on MCF-7 tumors grown in athymic nude mice.

### Effect of iodine on estrogen deprivation-induced tumor regression

Estrogen withdrawal-induced MCF-7 tumor regression in nude mice is well reported. We compared the sensitivity of topical Iodine Tincture treated and control MCF-7 tumors to estrogen deprivation. In ovariectomized athymic nude mice models, tumor growth from both control and iodine-treated group were regressed rapidly, while estrogen supplementation significantly stimulated tumor growth (Figure 5E). However, a slight difference in tumor growth was observed in the control and iodine-treated group. Tumors in the iodine treated mice regressed less than in the control group (Figure 5F–5G). Furthermore, the difference in tumor incidence at day 34 following tumor cell inoculation was also observed (Figure 5H). Among 18 tumor injections, only 7 measurable tumors were observed in control mice, while 11 measurable tumors were observed in the iodine-treated mice. Thus, in iodine treated mice where tumors regressed; the tumor shrinkage is less than that of control mice, which suggests an attenuating effect of topical application of iodine on tumor regression.

## DISCUSSION

The present study demonstrates a novel role for iodine in the context of stimulation of transcriptional activity of ER-α and ER-α-mediated growth of breast cancer cells. Iodine stimulates 1) ER-α mediated reporter transcriptional activity both in ER-positive and ER-negative but ER transfected breast cancer cells; 2) mRNA expression of ER-α regulated genes including *PR, PS2,* CyclinD1 and *Cat-D*; 3) protein expression of Cat-D, PR and CyclinD1; 4) an anchorage-dependent and –independent growth of ER-α positive breast cancer cells but not ER-α negative cells; and this stimulation is blocked by ER-α antagonist ICI 182,780; and 5) mRNA expression of *CycinD1* and *Cat-D* in MCF-7 xenograft in ovariectomized mice by topical application of Iodine Tincture. These data suggest a role of iodine as an ER-α agonist in stimulation of ER-α transcriptional activity and ER-α mediated growth of breast cancer cells.

Our results are in contrast to previous reports that iodine induces apoptosis of breast cancer cells [29–31]. The reasons for this inconsistency may relate to culture conditions, the concentrations of iodine, and the way of treatment. A high iodine concentration, especially in the absence of adequate free radical scavengers, may lead to the generation of excess free radicals [32, 33] and activation of a caspase-independent and mitochondria-mediated apoptotic pathway [29, 31, 34]. In present studies, the cells were treated with low dose iodine in combination with potassium iodide to maintain the sufficient molecular iodine concentrations and to reduce the influence of iodine volatilization. In addition, the iodine was presented to the cells cultured at low estrogenic conditions (stripped serum).

We provide clinical evidence demonstrating a significantly higher level of urine iodine in pre-menopausal breast cancer patients than that in normal health women (1012.5 ± 752.2 μg/g Cr vs. 235 ± 110.2 μg/g Cr). Individuals with urine iodine values > 500 μg/g Cr are considered to be actively taking high iodine foods [35]. Since the breast cancer patients in our studies were not consuming traditional high iodine foods or iodine supplements, we reasoned that their elevated levels of urine iodine more likely derived from the sources other than food. Absorption of iodine from topical skin application of Povidone-iodine (PVP-I) has been demonstrated in damaging skin [36], neonates [37], and those likely ophthalmic surgery patients [38]. We provide three lines of evidence indicating that the topical use of antiseptic PVP-I or Iodine Tincture prior to surgery is a major factor in elevated levels of urine iodine in breast cancer patients. First, a time-course analysis of urinary iodine levels of breast cancer patients undergoing breast surgery indicate that there is a sharp increase in iodine levels one day post-operation due to the topical use of antiseptic PVP-I and back to normal pre-operation levels at day 3 post-operation (Figure 4B). Second, this operation-related increase of urine iodine was also observed in the patients with endometriosis undergoing laparoscopic surgery (Figure 4C). Third, we demonstrated in the tumor xenograft nude mice model that topical application (skin rub) of Iodine Tincture results in an efficient systemic absorption of iodine and functional stimulation of estrogen-regulated genes in the MCF-7 tumor (Figure 5A–5D).

Although topical application of iodine doesn’t stimulate MCF-7 tumor growth, it attenuates tumors regression in the condition of estrogen deficiency. The effect of topical application of iodine on tumor growth is complicated. While iodine has estrogenic effects that may lead to stimulation of estrogen-dependent tumor growth, the broad spectrum antiseptic properties of iodine also imposes cytotoxicity, which leads to anti-proliferative and apoptotic effects on tumor growth. Whether topical application of iodine increases estrogen-dependent tumor growth depends on the balance of its estrogenic effect and antiseptic property, which might be related to iodine absorption and the local dose in the tumor microenvironment. Nevertheless, PVP-I is a water-soluble complex of iodine with a polymer and is a popular choice among surgeons all over the world. The estrogenic activity of iodine absorption from skin antiseptic use of PVP-I or Iodine Tincture should be considered, particularly for breast cancer patients prior to surgery and post-surgery follow up.

Our studies demonstrate that the estrogenic effect of iodine is significant in several folds. First, it has been reported that the uptake of ^125^I by transplanted hormone responsive mammary tumors in experimental mice is significantly higher, about 20 times greater than the uptake of ^125^I by hormone independent mammary tumors [39]. Second, although the correlation between the increased uptake of iodide and hormone dependency has not been demonstrated in humans, it has been demonstrated that the iodine transporter, sodium iodine symporter (NIS) is expressed in lactating mammary gland and mammary tumors [3, 4] but not in the unstimulated mammary gland. Immunohistochemical analyses also show that greater than breast cancer samples express NIS; in contrast, NIS was not detected in healthy breast tissue samples [4]. In fact, a case report indicated that ^131^I activity in thyroid cancer treatment was later found to accumulate in breast tissue [40]. Third, the NIS expression in breast cancer cells is associated with ER expression [25]. These studies suggest that the uptake of iodide is dependent upon the presence of hormone-dependent tumor cells within the tumor. However, the clinical relevance of NIS expression in breast cancer cells as well as its correlation with ER expression remains unknown. Our studies demonstrating the estrogenic effect of iodine provide a clue for a possible link between excess intake iodine and the increased incidence of breast cancer. It is noteworthy to emphasize that studies indicate a significantly elevated risk of developing breast cancer following thyroid cancer in pre-menopausal women [20, 41].

In conclusion, our study is the first to demonstrate that iodine has an estrogenic effect on stimulation of ER-α signaling on breast cancer cells *in vitro* and *in vivo*. Considering of the recent increase in iodine consumption, the present study may provide a possible clue for the increased incidence of breast cancer among the patients with thyroid cancer [21], and particularly, an increased breast cancer incidence among patients receiving ^131^I treatment. It is worthy to mention that iodine also affects estrogen metabolism by increasing estrogen metabolizing enzymes P450 1A1 (CYP1A1) and 1B1 (CYP1B1) [42, 43] the latter of which may lead to production of pro-carcinogenic and mutagenic 4-hydroxy estrogen metabolism [41, 43]. Thus we reason that iodine, under circumstances where it is able to generate excessive free radicals and ROS, may positively affect breast cancer incidence by enhancing an estrogenic environment on breast cancer cells.

## MATERIALS AND METHODS

### Reagent

Phenol red-free DMEM, RPMI 1640, charcoal-stripped fetal bovine serum (FBS), penicillin, streptomycin, and trypsin–EDTA solutions were supplied by Invitrogen (Carlsbad, CA, USA) and GIBCO-BRL (Grand Island, NY, USA). All chemical reagents were obtained from Sigma–Aldrich (St Louis, MO). RT reagent kit and Premix Ex Taq were from TAKARA (Kusatsu, Japan). Dual Luciferase Reporter Gene Assay Kit for detecting ER-α transcriptional activity was purchased from Promega (Madison, WI). Antibody for ER-α was got from Santa Cruz Biotechnology (Santa Cruz, CA). Primary antibodies for Cathepsin D (Cat-D), progesterone receptor (PR), PS2, GAPDH, β-tubulin, and all secondary antibodies were purchased from Abcam (Abcam, Cambridge, MA, USA).

### Assays for the transcriptional activity of ER-α

Cells were plated in 96-well plates for 24 h and transiently transfect with firefly luciferase reporter plasmid (pERE3-Luc). A renilla luciferase reporter plasmid, pRL-SV40-Luc, was used as an internal control for transfection efficiency. Lipofectamine2000 (Invitrogen) reagent was used for transfection. Cells, cultured in the conditioned phenol red-free DMEM medium containing 5% charcoal-stripped FBS for 3 days, were treated with or without different concentrations of iodine (1μM, 3μM and 6μM) and E2 for 24 h. Luciferase activities were measured using Dual Luciferase Assay System. Absolute ERE promoter firefly luciferase activity was normalized against renilla luciferase activity to correct for transfection efficiency. Triplicate wells were assayed for each transfection condition, and at least three independent transfection assays were performed.

### Real time PCR

E2 induced TFF1, PR, Cat-D and CyclinD1 genes expression were analyzed by quantitative real-time PCR (qPCR). The primer sequences (5′–3′) are as follows: TFF1, sense CCCCGTGAAAGACAGAATTGT, antisense GGTGTCGTCGAAACAGCAG; Cat-D, sense TGCTCAAGAACTACATGGACGC, Antisense CGAAGACGACTGTGAAGCACT; CyclinD1, sense GCTGCGAAGTGGAAACCATC, Antisense CATGGAGGGCGGATTGGAA; PR, Sense TTATGGTGTCCTTACCTGTGGG, Antisense GCGGATTTTATCAACGATGCAG; OAZ-1, Sense CGAGGACAGAGCCGCCTT, Antisense GACAAACCCAGGCGAGATGA.

### Soft agar colony formation assays

The anchorage-independent growth was carried out in 12-well plates as we previously described [44]. Briefly, the bottom layer consists of 0.5 ml of 5% charcoal-striped calf serum/IMEM containing 0.6% agar. The top layer consists of 0.25 ml of 5% charcoal-striped calf serum/IMEM containing 0.4% agar and approximately 2,000 cells. Cells were cultured under high humidity condition. Cells were fed with 0.1 ml of culture medium with or without different agents every four days. After two weeks, the number of colonies in each well was counted under a Nikon microscope at 100× amplification. Triplicate wells were assayed for each condition.

### Analysis of iodine from urinary samples

For clinical specimens from breast cancer patients, a total of 33 patients who have pathological diagnosis of breast cancer (prior to chemotherapy and hormone therapy) and 22 normal healthy women were involved. For women undergoing laparoscopic surgery for endometriosis, 8 patients with endometriosis and 8 normal healthy controls were involved. All participants were consuming a normal Chinese diet. None of the participants were using nutritional iodine supplements. After giving informed consent, urine samples were collected using the filter paper strip (Ahlstrom grade 226; ID Biological Systems), dried, stored at −20°C. Dried urine iodine extraction and analysis were conducted as we previously described [45]. For animal studies, nude mice were topically treated with Iodine Tincture every 2 or 3 day. Mouse urine samples were collected at the next day of iodine application.

### Tumor growth in athymic nude mice

A nude mouse tumorigenic assay was performed as we previously described [44]. Briefly, Approximately 5 × 10^6^ MCF-7 cells were injected into a 6-week-old female ovariectomized athymic nude mouse. Each animal received two injections, one on each side, in the mammary fat pads between the first and second nipples. For estrogen treatment, 17β-estradiol pellets (0.72 mg/pellet, 60-day releasing, Innovative Research of America, Toledo, OH) were implanted subcutaneously. For iodine treatment, mice were tropically applied with iodine by rubbing mouse back skin with Iodine Tincture (once every 2–3 day). Tumor size was determined every 2 or 3 days by three-dimensional measurements (in millimeters) using a caliper. Only measurable tumors were used to calculate the mean tumor volume for each tumor cell clone at each time point.

### Statistical analysis

The data were expressed as mean ± S.D. Differences between experimental groups were analyzed using a one-way ANOVA and Tukey’s test, and the urinary iodine concentration was compared using unpaired *t*-test. differences with *P* < 0.05 were considered statistically significant. All mean values were calculated from at least three repeated experiments.

## Abbreviations

ER-α: estrogen receptor-α
UIC: urinary iodine concentration
RAI: radioactive iodine
NIS: sodium/iodide symporter
qPCR: Quantitative real time polymerase chain reaction
PVP-I: Povidone-iodine
